# Differentiation therapy and the mechanisms that terminate cancer cell proliferation without harming normal cells

**DOI:** 10.1038/s41419-018-0919-9

**Published:** 2018-09-06

**Authors:** Francis O. Enane, Yogen Saunthararajah, Murray Korc

**Affiliations:** 1 Department of Medicine, Indiana University School of Medicine Indianapolis, Indianapolis, IN 46202 USA; 2 Department of Hematology and Oncology, Taussig Cancer Center, Cleveland Clinic, Cleveland, OH 44195 USA; 3 Department of Translational Hematology and Oncology Research, Taussig Cancer Center, Cleveland Clinic, Cleveland, OH 44195 USA; 4 Department of Biochemistry and Molecular Biology, Indiana University School of Medicine, Indianapolis, IN 46202 USA; 5 The Pancreatic Cancer Signature Center at Indiana University Purdue University Indianapolis and Indiana University Simon Cancer, Indianapolis, IN 46202 USA

## Abstract

Chemotherapeutic drugs have a common intent to activate apoptosis in tumor cells. However, master regulators of apoptosis (e.g., p53, p16/CDKN2A) are frequently genetically inactivated in cancers, resulting in multidrug resistance. An alternative, p53-independent method for terminating malignant proliferation is to engage terminal-differentiation. Normally, the exponential proliferation of lineage-committed progenitors, coordinated by the master transcription factor (TF) MYC, is self-limited by forward-differentiation to terminal lineage-fates. In cancers, however, this exponential proliferation is disengaged from terminal-differentiation. The mechanisms underlying this decoupling are mostly unknown. We performed a systematic review of published literature (January 2007–June 2018) to identify gene pathways linked to differentiation-failure in three treatment-recalcitrant cancers: hepatocellular carcinoma (HCC), ovarian cancer (OVC), and pancreatic ductal adenocarcinoma (PDAC). We analyzed key gene alterations in various apoptosis, proliferation and differentiation pathways to determine whether it is possible to predict treatment outcomes and suggest novel therapies. Poorly differentiated tumors were linked to poorer survival across histologies. Our analyses suggested loss-of-function events to master TF drivers of lineage-fates and their cofactors as being linked to differentiation-failure: genomic data in TCGA and ICGC databases demonstrated frequent haploinsufficiency of lineage master TFs (e.g., GATA4/6) in poorly differentiated tumors; the coactivators that these TFs use to activate genes (e.g. ARID1A, PBRM1) were also frequently inactivated by genetic mutation and/or deletion. By contrast, corepressor components (e.g., DNMT1, EED, UHRF1, and BAZ1A/B), that oppose coactivators to repress or turn off genes, were frequently amplified instead, and the level of amplification was highest in poorly differentiated lesions. This selection by neoplastic evolution towards unbalanced activity of transcriptional corepressors suggests these enzymes as candidate targets for inhibition aiming to re-engage forward-differentiation. This notion is supported by both pre-clinical and clinical trial literature.

## Facts

- Treatment outcomes for most disseminated p53 mutant solid tumors are poor.
- The most lethal of these tumors are morphologically poorly differentiated.
- Differentiation-restoring treatments are an emerging non-toxic, p53-independent treatment alternative.
- Advances in omics big data can be used to define molecular targets for differentiation-restoring therapy.
- Pharmacological inhibition of corepressor enzymes re-engages suppressed differentiation pathways.

## Open questions

- Which among the multiple corepressors identified in cells are rational molecular targets for pharmacologic manipulation?
- What is the in vivo evidence of how corepressor inhibiting therapy triggers terminal-differentiation?
- What are the key MYC-antagonists in any given lineage that mediate cell cycle exits by terminal-differentiation?

## Introduction

Conventional chemotherapy aims to activate apoptosis even in tumors where master regulators of apoptosis are physically unavailable through inactivating gene mutations, leading to multi-drug resistance^1^. Therefore, alternative targets and pathways of therapy are needed. The complex process of cellular proliferation is coordinated by the master transcription factor (TF) MYC. Master TF that drive commitment into a lineage cooperate with MYC to drive exponential proliferation, but simultaneously drive forward-differentiation that culminates in cell cycle exits^2–4^. Malignant exponential self-replication involves decoupling of proliferation from this forward-differentiation^5–7^. One way of approaching the mechanisms underlying such decoupling is to consider three major modules in multi-cellular cell physiology: (i) proliferation or replication; (ii) apoptosis; (iii) lineage-differentiation^7^. Cell proliferation, the essence of all cancers, is coordinated by the master TF MYC^2,8,9^—amplification of the *MYC* gene, and activation of MYC or its paralogues is seen across all human malignancies^10^. The master regulator of cellular apoptosis p53 (*TP53*) or its key cofactors are almost universally inactivated in human malignancies^11^—while multiple copies of TP53 protect elephants from a high rate of cancer proportionate to their high cellular mass^12^, *TP53* mutations can be viewed as “the elephant in the room” of all cancer therapy, since most treatments intend to activate this master regulator which ironically is absent from most cancers. The mechanisms underlying impeded lineage-differentiation in cancer are still mostly opaque and require wider mechanistic characterization to allow development of therapeutic interventions aiming to restore lineage-fates.

Differentiation-failure is used to distinguish malignant from benign tumors^13^, and the degree of differentiation-failure separates high from low aggressive transformations, e.g., pancreatic intraepithelial neoplasia (PanIN) lesions from pancreatic ductal adenocarcinoma (PDAC). Loss-of-differentiation may not be obvious by light microscopy, but can be detected using gene expression analysis of differentiation factors^6^. Tissue differentiation is usefully considered in three compartments: (1) tissue stem cells—cells with an intrinsically low proliferation rate but capable of self-renewal and of giving rise to daughter cells committed into various tissue-lineages^14–16^; (2) lineage-committed progenitors—cells with high levels of MYC activity^17–20^ and exponential proliferation that is coupled with forward differentiation towards lineage-fates; and (3) terminally differentiated cells that have transitioned from exponential proliferation to a focus on performing specialized tissue functions^21–23^. Each stage of differentiation is regulated by key master TFs—stem cell TFs, lineage-progenitor TFs and terminal-differentiation TFs. The purpose of this review and study is to define differentiation related molecular targets that can be used in the development of p53 independent therapies that are not toxic toward normal stem cells and that do not alter normal stem cell replication.

## Methods

We conducted a systematic review in accordance to PRISMA guidelines (Fig. S1)^24^. We searched the national library of medicine through PubMed for literature containing cell proliferation, apoptosis, and differentiation in cancer (Fig. S1). Search terms included chemotherapy, cancer apoptosis, proliferation, and cell differentiation. We also searched the work cited in the identified articles for additional relevant literature. We then focused on three therapy-resistant cancers: Hepatocellular carcinoma (HCC), Ovarian cancer (OVC), and pancreatic ductal adenocarcinoma (PDAC). Additional terms of phase 1, 2, and 3 randomized clinical trials were searched in Web Science, Pubmed/MEDLINE, Embase, ClinicalTrials.gov and Google Scholar. The search strategy included studies published in English language from January 2007 to June 2018.

We then analyzed gene datasets from The Cancer Genome Atlas (TCGA) (https://cancergenome.nih.gov/) and International Cancer Genome Consortium (ICGC) (http://icgc.org/) to eliminate risk of bias such as selective reporting and publication bias of altered pathways. Search terms for altered genes in both databases were mut (missense, frameshift, inframe, truncating mutations), Hetloss (heterozygous deletion), Homdel (homozygous deletion), gain, and amplification^25,26^. We further searched protein–protein interactions in literature and in data deposited in UniProt (http://www.uniprot.org/) to identify master TFs and their interacting partners necessary for gene activation (coactivators) or repression (corepressors) (Table 1).

**Table 1:** Lineage specific master transcription factors, coactivators and corepressors of various tissues and identified genetic alterations in human malignancies

Tissue (n - TCGA)	Master transcription factor	Alteration frequency in TCGA database	Uniprot predicted coactivators	Alteration frequency in TCGA database	Uniprot predicted corepressors (http://www.uniprot.org/)	Alteration frequency in TCGA database
Liver (*n* = 442)	*GATA4* ^6, 119^	67% Hetloss	*ARID1A*	44% Hetloss, fs*	KDM1B	42% Amp, Gain
			*ARID2*	17% Hetloss, fs*	BAZ1B	
	*FOXA1* ^120^	3% Amp Gain	*KMT2A*	28% Hetloss, fs*	SUZ12	32% Amp, Gain
			*SMARCA4*	24% Hetloss, fs*	DNMT1	
	*FOXA2* ^120^	30% Amp Gain	*SMARCAD1*	46% Hetloss	BAZ2A	25% Amp, Gain
						15% Amp, Gain
						14% Amp, Gain
Pancreas (*n* = 109)	*GATA4* ^121^	49% Hetloss	*ARID1A*	49% Hetloss, fs*	BAZ1B	57% Amp, Gain
			*ARID1B*	61% Hetloss	DNMT1	
	*GATA6* ^121^	23% Hetloss	*ARID3C*	43% Hetloss	UHRF1	50% Amp, Gain
			*SMARCD1*	50% Hetloss	SUZ12	
	*PTF1A* ^122^	20% Hetloss	*SMARCB1*	34% Hetloss	BAZ2A	45% Amp, Gain
	*FOXA2* ^123^	43% Amp Gain				40% Amp, Gain
	*PDX1*	58% Hetloss				39% Amp, Gain
Ovary (*n* = 302)	*GATA4* ^83^	69% Hetloss	*ARID3A*	91% Hetloss	EZH2	38% Gain amp
			*ARID3B*	50% Hetloss	DNMT1	39% Gain amp
	*FOXL1/2* ^82, 87^	67% Amp, Gain	*ARID3C*	37% Hetloss	BAZ1A	18% Gain amp
			*SMARCAD1*	68% Hetloss	EED	39% Gain amp
	*FOXO1* ^124^	63% Hetloss	*ARID1B*	67% Hetloss		
			*ARID1A*	44% Hetloss		
			*SMARCA1*	47% Hetloss		

We analyzed TCGA data in cBioPortal to determine genetic alterations in genes mediating differentiation pathways. Lineage specific transcription factors were identified using lineage tracing studies. Cofactors interacting with lineage specific transcription factors were determined using data deposited in UniProt database (http://www.uniprot.org/). Master transcription factors are lineage specific and they recruit various coactivators to cooperate and turn on differentiation genes. While heterozygous loss of GATA4 and inactivation by frameshift mutations of GATA4 coactivators are frequent in hepatocellular carcinoma, other master transcription factors such as FOXA1 are available to mediate differentiation pathways. However, corepressors such as KDM1B, which are also recruited by these TFs, are aberrant in HCC by copy number gains and amplification. Such alterations impair ability for differentiation to ensue in HCC through epigenetic suppression of target genes^5, 88, 89^. These forms of alterations are commonly observed also in PDAC, and OVC. Since corepressors are either gained or amplified in cancer but not inactivated by frameshift mutations, inhibition of these enzymes may serve as logical molecular targets of therapy (Fig. 5c, d)

*Frameshift mutation; hetloss, heterozygous deletion

## Results

### MYC amplification decreases survival across multiple human malignancies

**Fig. 1:**
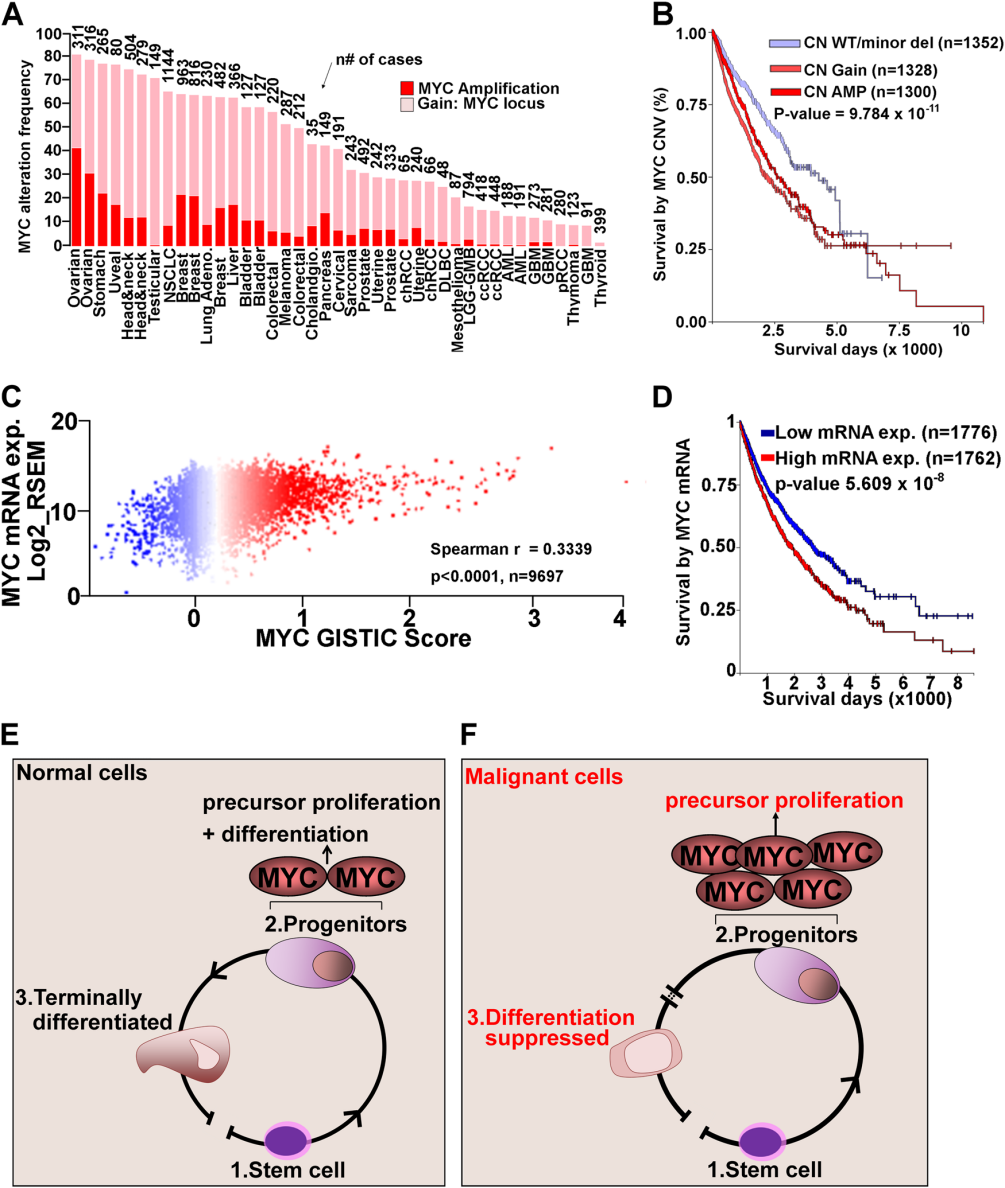
MYC alterations across multiple human malignancies. **a** TCGA and IGCG data were analyzed through cBioPortal to determine aberrations at the MYC locus using pre-assigned GISTIC scores in multiple cancers from different tissue types. **b** We analyzed TCGA PANCAN data sets available through TCGA hub in Xena Browser. Survival analysis of cases with copy number (CN) gains and amplification at the MYC loci vs. those with CN WT/minor deletion of MYC demonstrated a significant overall survival (*p*-value < 9.784E−11, LogRank test, *n* = 1352 WT/minor del, 2628 CN gain and amplification). Survival data analyzed in Xena Browser (https://xenabrowser.net/) **c** Anlysis of MYC GISTIC Score vs. MYC mRNA expression using PANCAN RNA-seq data available in TCGA hub in Xena Browser. There was a strong correlation with spearman *r* = 0.3339, *p* < 0.0001, *n* = 9697. **d** Survival analysis of patients with increased MYC mRNA compared to those with decreased MYC mRNA expression. Expression levels are normalized relative to expression levels in normal tissues. Increased MYC mRNA was associated with poor survival (*n* = 1762) compared to decreased MYC mRNA (*n* = 1776, *p* = 5.06 × 10^−18^ **e** Schematic representation of metazoan differentiation and how differentiation is stalled in malignant cells. Differentiation continuum is initiated through stem cells lineage commitment, followed by exponential proliferation of tissue precursors/progenitors mediated by two copies of the MYC gene. To maintain homeostasis, MYC-mediated proliferation is dominantly antagonized by terminal differentiation pathways. **f** Human malignancies have impaired differentiation that fails to antagonize the MYC gene allowing for exponential proliferation of tissue precursors

One of the key TFs regulating mammalian cell proliferation is the myelocytomatosis viral oncoprotein (MYC), whose function is conserved across evolutionary hierarchies^27–31^. Physiologically, MYC regulated proliferation is succeeded by lineage-differentiation programs that antagonize MYC to terminate proliferation^20^. We analyzed *MYC* alterations by two approaches. First, we analyzed copy number (CN) alterations at the *MYC* locus using TCGA and ICGC data available through cBioPortal platform and found frequent amplifications and gains of *MYC* (Fig. 1a). We then accessed TCGA pan-cancer (PANCAN) data containing 11,000 patients across 33 of the most prevalent tumors and analyzed it through Xena Browser. *MYC* was highly amplified across these malignancies^8,10^. In both data sets *MYC* CN changes were determined using GISTIC score method, where values of −2,−1,0,1,2, represented homozygous deletion, heterozygous deletion, diploid, low-level amplification, or high-level amplification^32^. We next performed survival analysis using GISTIC scores predicting low level deletion/wild-type *MYC*, vs. gain/amplification using the PANCAN dataset. *MYC* amplification correlated with decreased overall survival (*p* < 9.784 × 10^−11^, *n* = 2628) compared to cases with *MYC* CN WT/low level deletions (*n* = 1352) (Fig. 1b). We then analyzed the correlation between GISTIC scores at the *MYC* locus vs. *MYC* mRNA expression and patient survival. There was a strong correlation (spearman *r* = 0.3339, *p* < 0.0001, *n* = 9697) between *MYC* GISTIC score and MYC mRNA expression (Fig. 1c). High (*n* = 1762) vs. low *n* = 1776) MYC mRNA levels were associated with decreased (*p* < 5.609 × 10^−8^) overall survival (Fig. 1d). Thus, *MYC* is a vital oncogene across many human malignancies and identification of mechanisms to antagonize MYC in cancer could have therapeutic applications. MYC function is conserved across evolutionary hierarchies^27–31^. The simple life cycle of protozoa requires MYC to generate daughter cells that resemble their parental cells with each cell division^27,29^. Evolution from single cell organism to multicellular organisms led to intense use of energy to open the chromatin and to expose naked DNA allowing lineage TFs to bind and activate hundreds of terminal differentiation genes that guide cell fate and specialization into various layers of cells. This process does not require actively proliferating cells. Hence, MYC mediated proliferation is potently antagonized at this stage^33,34^ (Fig. 1e). This form of potent MYC antagonism is also necessary for the existence of multi-cellularity^29,35^. Convincingly, infection of multicellular organisms with protozoa parasites enhances transformation of infected cells into proliferative cells by complex mechanisms that activate MYC protein and suppress differentiation TFs^27,29,36^.

Unlike normal cells, malignant cells undergo proliferation without terminal-differentiation (Fig. 1e, f). This aberrant process is strongly dependent on stabilization of MYC and its co-proteins that modulate cell growth and division^17,20,37–39^. Genetic and epigenetic alterations ensure that persistent proliferation of lineage committed progenitors occurs without final differentiation in cancer cells (Fig. 1e)^7^. First, persistent proliferation is achieved by consistent upregulation and chromosome gains of the genetic locus encoding the *MYC* gene across human malignancies (Fig. 1a). *MYC* amplification predicts poor overall survival (LogRank *p*-value = 9.784 × 10^−11^, *n* = 3980) (Fig. 1a, b). In studies using genetically engineered mouse models (GEMM) or xenograft models of cancer, antagonizing MYC sustains tumor regression across multiple tumors^39–41^. For instance, Shachaf et al. developed a transgenic mouse model conditionally expressing MYC in hepatocytes using tetracycline-controlled expression^39^. Inactivation of Myc induced regression of murine HCC increasing hepatocytes and hepatobiliary cell differentiation, loss of HCC marker α-fetoprotein, and suppressed proliferation^39^. In a xenograft PDAC model, Zhang et al. targeted MYC-MAX dimerization with a small molecule (10058-F4) that disrupts the MYC transcriptional activity^40^. Addition of 10058-F4 to gemcitabine led to drastic attenuation of tumorigenesis compared to single agent treatment^40^. Using a Kras driven mouse model of lung cancer, Soucek et al. targeted MYC using a dominant negative MYC dimerization domain mutant disrupting MYC binding to canonical Myc E-box response element ‘CACGTG’, thereby inhibiting MYC transactivation activity^41^. Inhibition of MYC transactivation increased mice survival by terminating lung cancer growth^41^.

From a translational perspective, various challenges exist in the attempt to directly target MYC pharmacologically^42^. The most important challenge is that proliferation is a feature of normal progenitors and such therapy could have a poor therapeutic index^20^. Additionally, tumors have heterogeneous genetic backgrounds contributing to sustained MYC activity. Therefore, to understand mechanisms that antagonize excessive MYC actions, it is imperative to define the evolutionary conserved physiological methods by which normal progenitors antagonize MYC to turn off intense proliferation and how these can be restored in cancer.

### Terminating proliferation by engaging apoptosis is toxic to normal dividing cells

To retain cohesion and integrity between different cell types, multicellular organisms have evolved a system of checks and balances collectively known as apoptosis^43,44^. The master TFs of apoptosis p53 (*TP53*) and its cofactor p16 or p14ARF (*CDKN2A*) play crucial roles by arresting proliferating cells to enable repair of damage, or initiating orderly suicide if such damage cannot be repaired^45,46^. During embryogenesis, expression of p53 is downregulated perhaps because embryonic stem cells self-renew without exponentially proliferating^47–49^. Functional studies of differential expression of p53 using reporter assays demonstrated higher expression at later developmental stages, and decreased expression in terminally differentiated cells^48^. During cell division, p53 pathways potently antagonize MYC pathways to halt proliferation allowing impaired cells to undergo repair; irreparable cells undergo self-destruction through irreversible apoptosis to protect the integrity of entire organism^43^. Since p53-knockout (KO) mice have normal development and are not enlarged^50^, this illustrates that apoptosis pathways are not the dominant mechanisms used by lineage-progenitors to terminate exponential proliferation. Thus, mice exhibiting double KO of *Trp53*, and Phosphatase and tensin homolog (*Pten*) develop glioma tumors by failing to antagonize MYC, but this phenotype is only observed in the *Trp53* and *Pten* double knockouts^45,46^. In PDAC the most frequent gene mutation is *KRAS* (~92%). GEMMs in which mutant *KRAS* (KC mice) is expressed in pancreas cells develop PDAC in 30 to 40% of cases at ~8–12 months of age^51^. Adding mutant *Trp53* to the above GEMM (KPC mice) increases PDAC penetrance and decreases survival to ~5 months whereas KC mice with *Ink4a* deletion survive for ~2–3 months^52,53^. Mice with mutant *Trp53* alone without mutant Kras do not develop PDAC^53^. By contrast, in ovarian cancer mouse models it has been demonstrated that *Trp53* inactivation results in invasive tumors but tumor development is accelerated in mice with concomitant inactivation of *Brca1* and *Trp53*^54^.

**Fig. 2:**
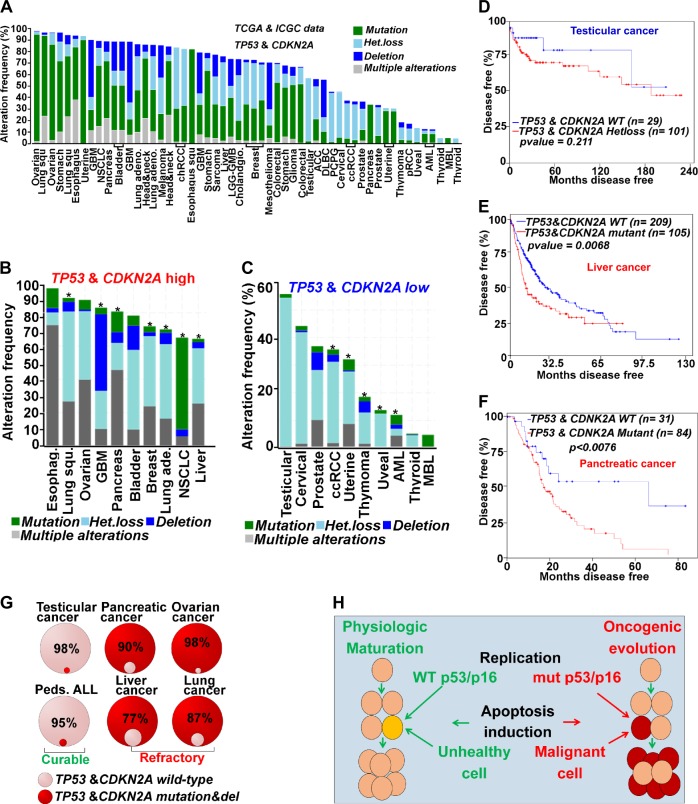
Apoptosis induction in p53/p16 mutant malignancy remains toxic to normal cells while simultaneous linked to refractory disease. **a** Data was downloaded from TCGA and ICGC and analyzed in cBioPortal for mutations in *TP53* and *CDKN2A* genes. **b** Top 10 malignancies with high TP53/CDKN2A alterations (TP53/CDKN2A high). *Cases where these alterations were linked to poor disease-free or overall survival with a *p*-value < 0.05 (Table S1). **c** Bottom 10 cases with least frequency of alterations in TP53/CDKN2A (TP53/CDKN2A low). *Cases where these alterations were linked to poor disease-free or overall survival with a *p*-value ≤ 0.05 **d** Disease-free survival of testicular cancer, cases with minor alterations (gains, and heterozygous loss of one allele in *TP53* and *CDKN2A*) vs. cases with wild type *TP53* and *CDKN2A* (*p*-value = 0.211, LogRank test). **e** Disease-free survival of pancreatic cancer with mutant *TP53* and *CDKN2A* cases was significantly lower vs. cases with wild-type *TP53* and *CDKN2A* (*p*-value = 0.0078, LogRank test). **f** Disease-free survival of liver cancer was also significantly lower in mutant *TP53* and *CDKN2A* cases vs. wild-type (*p*-value = 0.0068, LogRank test). **g** Quantitative analysis of *TP53* and *CDKN2A* mutations demonstrated less frequency of alteration of these genes in curable vs. high refractory/treatment resistant human malignancies. **h** During physiologic maturation, unhealthy cells with WT p53/p16 undergo irreversible apoptosis. Alterations in these proteins sustain oncogenic evolution leading to aberrant proliferation without apoptosis

*TP53* and *CDKN2A* are frequently bi-allelically inactivated across human malignancies (Fig. 2a). Such inactivation has major impact on treatment^7^. To terminate malignant proliferation, conventional chemotherapeutics aim to upregulate p53/p16 by inducing cytotoxic stress that mimics physiological activators of this pathway^55^. Since malignant cells and normal cells co-exist within the same milieu, such treatment has an unfavorable therapeutic index, as these genes are mutated/physically unavailable in malignant cells, but intact in normal cells. Multiple methods to re-engage apoptosis in cancer therapy have been investigated but it has been difficult to address this fundamental issue of therapeutic index^56^. Advances in genomic techniques indicate that when *TP53*/*CDKN2A* genes are wild-type, as in testicular cancer, treatment with cytotoxic chemotherapy (e.g., cisplatin) produces complete responses that increase overall and disease-free survival^57^ (Fig. 2a, b). Malignancies with high rates of *TP53*/*CDKN2A* inactivation do not exhibit these responses leading to resistance to multiple apoptosis-based treatments (broad chemo-resistance and radio-resistance) (Fig. 2a, b, e, f)^7^. Even different tumor types originating from the same organ have better responses to therapy if apoptosis genes are intact. For instance, *TP53* and *CDKN2A* mutations occur in ~70 and 90% of PDAC, respectively^58^ (Fig. 2a). The overall 5 year survival rate in PDAC is ~9% even when including patients treated with chemotherapy or combination therapies and/or surgery^59,60^. By contrast, pancreatic neuroendocrine tumors (PNETs), generally do not harbor *TP53* mutations, exhibit only minimal deletions of *CDKN2A*^61^, and have a 5 year survival rate of >50% when treated with apoptosis-inducing therapy^62^. Similarly, glioblastoma multiform (GBM) exhibits a variety of clinical, histopathologic, and molecular characteristics, and harbor *TP53* mutations in ~30% of primary cases and ~65% of secondary GBM^63,64^. Glioma cells with WT *TP53* are responsive to cytotoxic stress induced by clinically available chemotherapeutic agents compared to those with transcriptionally silenced mutant *TP53*^65–67^. Additionally, in the *Trp53* induced mouse model of PDAC (KPC), genetic inactivation of one allele of *Myc* sensitizes therapeutic response gemcitabine^40^. We therefore analyzed genomic data by comparing the top ten malignancies with an elevated frequency of *TP53/CDKN2A* alterations (*TP53/CDKN2A*-high) with the bottom ten malignancies with low-frequency *TP53/CDKN2A* alterations (*TP53/CDKN2A*-low) (Fig. 2b, c). We found that 7/10 of *TP53/CDKN2A*-high cancers had a decrease in disease-free and overall survival when these genes were mutated (Fig. 2b; Table S1) (*p*-values < 0.05). Consistently, even in *TP53/CDKN2A*-low cases, there was a decrease in disease-free and overall survival when these genes were altered (*p*-values < 0.05) (Fig 2c; Table S1). Thus, the rate of alterations in apoptosis genes is lower in curable malignancies (testicular cancer/pediatric ALL) compared to high refractory/treatment resistant cancers (PDAC/HCC) (Fig. 2g). During physiologic maturation, WT *TP53* induces irreversible apoptosis of unhealthy cells to retain integrity of entire organism (Fig. 2h). By contrast, oncogenic evolution mutates mediators of apoptosis leading to resistance to apoptosis induction (Fig. 2h).

### Genetic and epigenetic alterations of differentiation genes in cancer

The most aggressive human malignancies are poorly differentiated^13^. While differentiation contributes to poor survival across multiple human malignancies, the mechanisms that underpin differentiation impediment in malignant cells are mostly unclear, but new knowledge is emerging^5–7^. We identified key lineage master TFs for the development of the ovary, pancreas and liver using published lineage conversion studies, or studies with transgenic mouse models^6,68–74^ (Table 1). Cellular differentiation and lineage commitment programs are dictated by this handful of master TFs and their cofactors. While multiple cofactors have major roles, the most vital of these are transcriptional coactivators and corepressors that use ATP to remodel chromatin to turn-on or turn-off target genes^33,34,75^. Accordingly, we analyzed the genetic alterations in lineage TFs, their coactivators and corepressors in OVC, PDAC, and HCC (Table 1).

**Fig. 3:**
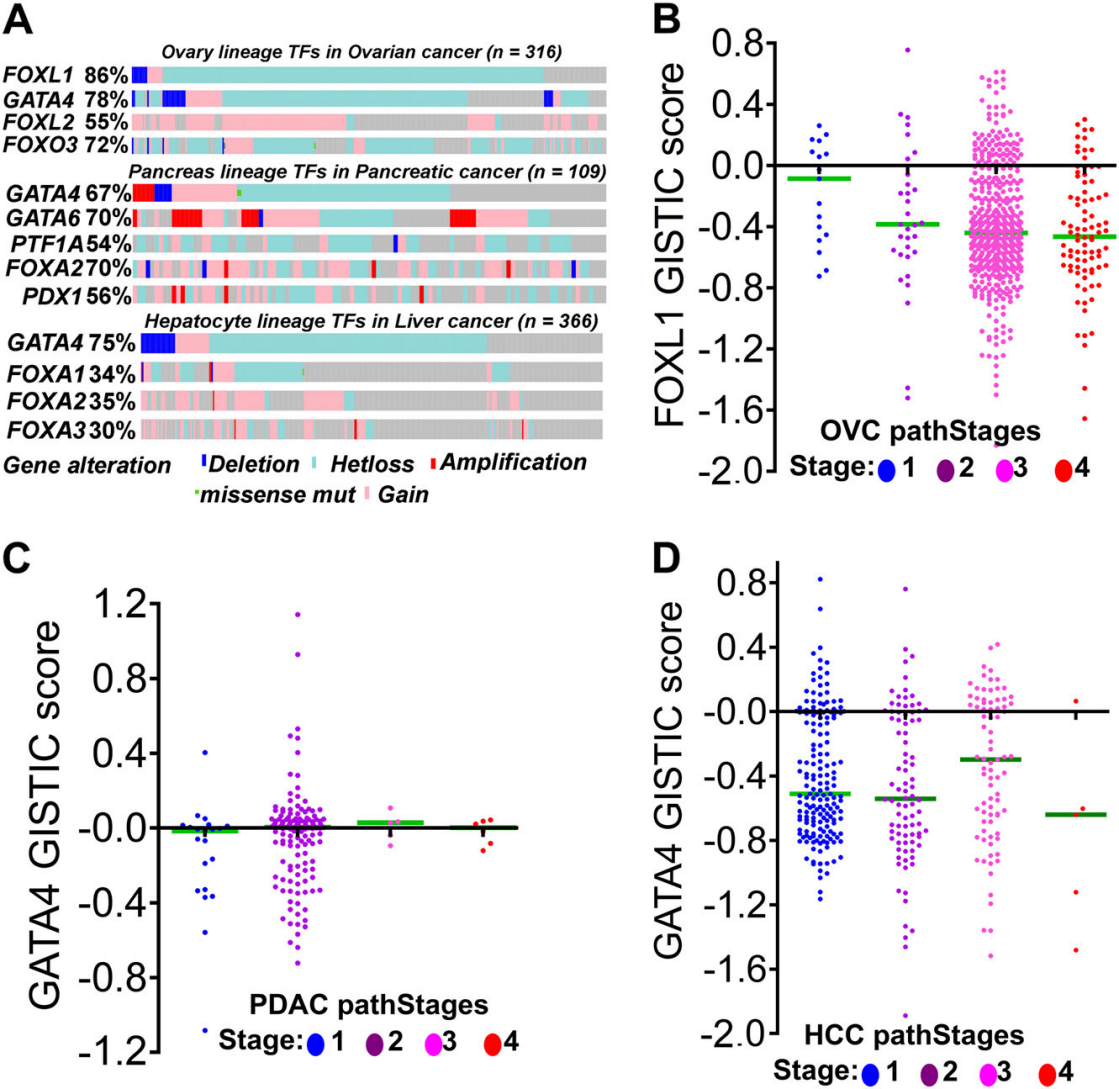
Genetic alterations in lineage specifying master transcription factors in human malignancies. **a** Analysis of TCGA data deposited in cBioPortal to determine alterations of master transcription factors of various lineages (Table 1). Key lineage specifying transcriptions factors were mostly haploinsufficient (heterozygous deletion/hetloss) in malignant cells or contained frequent amplification and gains. None of the transcription factors had biallelic frameshift inactivating mutations. Thus stalled differentiation occurs through genetic haploinsuffiency of key lineage specific transcription factors^6^. **b** Analysis of FOXL1 deletions across varying degrees of differentiation grades (pathological grades) of ovarian cancer. **c** Analysis of GATA4 deletions across varying degrees of differentiation grades of pancreatic cancer (PDAC). **d** Analysis of GATA deletions across varying degrees of differentiation grades liver cancer (HCC)

**Fig. 4:**
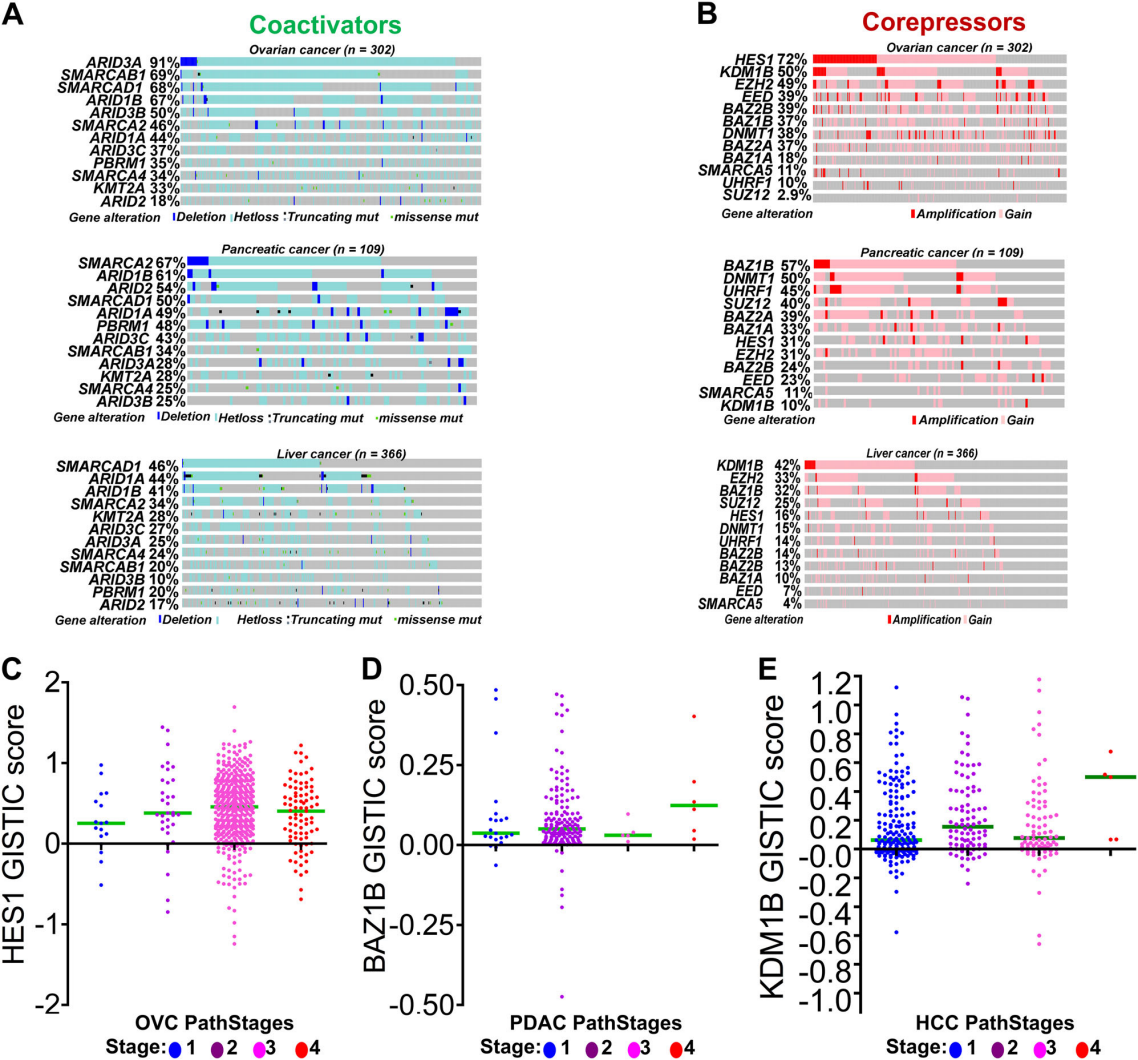
Frequent inactivating mutations of coactivators and amplification and copy number gains at gene loci of transcriptional corepressors. TCGA data was analyzed in cBioPortal to determine frequent genetic alterations in transcriptional corepressor and coactivator enzymes (Table 1). **a** Inactivating mutations, bi-allelic and frameshift mutations and deletions of transcriptional coactivator enzymes in ovarian, pancreatic and liver cancers (Table 1). **b** Copy number (CN) gain and amplifications of corepressors was frequently observed in various tumors including ovarian cancer (OVC), pancreatic cancer (PDAC) and liver cancer (HCC) (Table 1). **c** Analysis of HES1 CN gains across varying degrees of differentiation grades (pathological grades) of OVC. **d** Analysis of CN gains of BAZ1B across varying degrees of differentiation grades PDAC. **e** Analysis of CN gains KDM1B gains across varying degrees of differentiation grades HCC

Since malignant cells cannot completely suppress differentiation, as it is a continuum along which all cells exist, master TFs that specify commitment into various lineages are nearly never completely inactivated by mutation but are frequently haploinsufficient (Fig. 3a; Table 1). This dose-reduction is sufficient to stall advances along the differentiation continuum at its most proliferative points^5–7^. For instance, FOXL1 loss was frequent in OVC (Fig. 3a), and the frequency of *FOXL1* loss was highest in poorly differentiated OVC (Fig. 3b). This pattern was similar for *GATA4* in PDAC and HCC, even though these malignancies had small numbers of patients surviving beyond stages I and II (Fig. 3b, c). We identified key interacting partners that are coactivators and corepressors of various lineage specific TFs (Table 1) by literature analysis and data deposited in UniProt database (http://www.uniprot.org/). To augment the stalled differentiation, the coactivators were found frequently inactivated and deleted (Table 1; Fig. 4a) favoring repression of downstream genes targeted by key TFs. New lines of evidence now imply that such alterations impair pathways mediating terminal differentiation^6,7,76^. Early discoveries of the functions of these coactivator enzymes demonstrated that their role in physiology was to utilize ATP to mobilize histone DNA interactions such that naked DNA was exposed, thereby allowing TFs to bind to and activate target genes^33,34,75,77^. This process is conserved in evolution from yeast^78^, one of the simplest metazoa, to *homo-sapiens*^77^. Inactivation of these genes in cancer could be an attempt to impair the ability of coactivators to expose DNA to master TFs that activate downstream genes. A major clue to this hypothesis is that lineage master TFs are selective in their use of specific coactivators to mediate activation of lineage genes (Table 1). Another clue is that malignant cells tend to lose one allele of lineage specifying TFs, an event that may be sufficient to allow lineage commitment but insufficient for terminal differentiation^6,7^ (Fig. 3a; Table 1). For instance, liver progenitors require cooperation between GATA4 and FOXA1 to recruit coactivators (e.g., ARID1A) and mediate activation of hepatocyte differentiation genes. In HCC, heterozygous loss of *GATA4* is frequent (68%, *n* = 366, Fig. 3a; Table 1) and inactivating mutations in *ARID1A* are common (44%, *n* = 366, Fig. 4a; Table 1)^6^. Hepatic differentiation is impaired and proliferation enhanced in livers with *Gata4* or *Arid1a* liver-conditional haploinsufficiency^6,76,79^. Moreover, reintroduction of GATA4 in *GATA4* deficient HCC, or ARID1A in *ARID1A* mutated but GATA4 intact HCC, activates hundreds of hepatocyte epithelial-differentiation genes^6^. The master TFs of the pancreatic lineage include GATA4 and GATA6^80,81^. Copy number losses of one allele of these factors are seen in PDAC, with loss of function mutations in coactivators also observed (Table 1; Figs. 3a, 4a). However, PDACs also exhibited a high incidence of amplification or gain of *GATA4* and *GATA6*, suggesting that in certain instances these TFs may confer a growth advantage to pancreatic cancer cells. In OVC, one allele of ovarian master TFs *FOXL1*^82,83^ is frequently lost (80%, Fig. 3a; Table 1 *n* = 316), while coactivators, such as ARID3A and ARID3B, are often inactivated (Table 1; Fig. 4a). Thus at the core of malignant transformation, differentiation impediment routinely enhances malignant proliferation and is achieved through haploinsufficiency of master TFs and inactivation of the coactivators they use. This understanding could lead to treatments aiming to re-engage forward-differentiation, as an alternative to apoptosis, as the means of terminating malignant proliferation.

### Corepressor enzymes: emerging targets for differentiation-restoring oncotherapy

An enhanceosome is composed of multiprotein complexes cooperating to activate genes of a given lineage^84,85^, e.g., hepatic enhanceosomes activate hepatocyte genes^6^, whereas pancreas and ovarian enhanceosomes activate pancreatic^86^ and ovarian genes^87^, respectively. Genetic disruption of this cooperation can shift the content of these protein hubs away from coactivators to corepressors that repress lineage genes instead^76,88,89^. Such repression is further enabled by the inherent closed chromatin status of terminal-differentiation genes, contrasting with inherently open chromatin at proliferation and early-differentiation genes^6,7,90^.

**Fig. 5:**
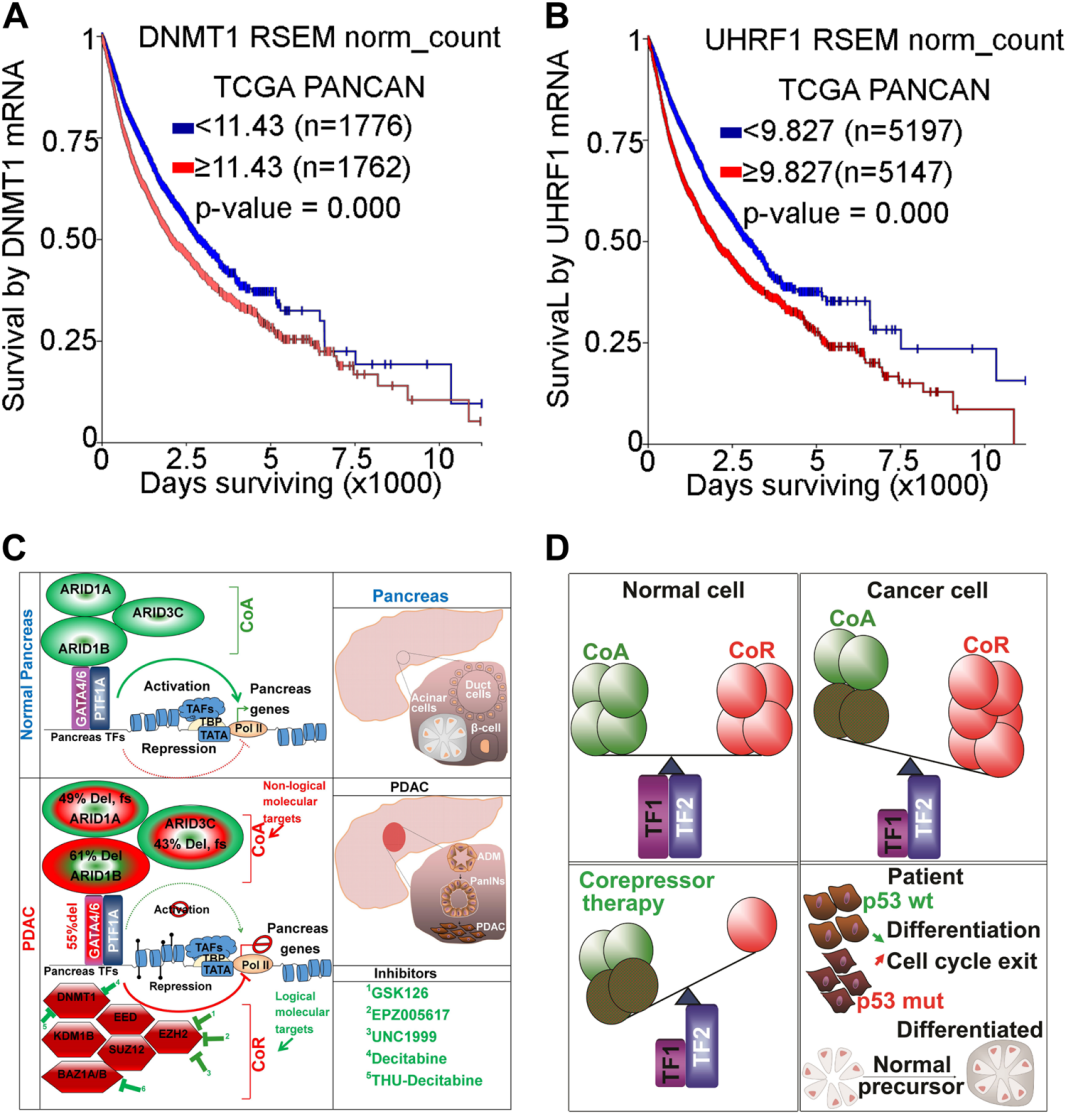
Corepressor upregulation and model for inhibiting corepressors to re-engage forward-differentiation. **a** Corepressor DNMT1 mRNA upregulation predicts poor survival across multiple human malignancies in TCGA PANCAN data. **b** Corepressor UHRF1 (that partners with DNMT1 for epigenetic repression activities) mRNA upregulation predicts poor survival across multiple human malignancies in TCGA PANCAN data. **c** Model example in PDAC alterations of coactivators and corepressors and candidate small molecules that can be used as corepressor therapy. **d** Model schematic summary for p-53 independent differentiation-restoring therapy. Non-malignant cells (normal cells) have intact lineage specifying transcription factors of cell fate determination that dynamically recruit coactivators and corepressors enzymes to turn on or turn off differentiation genes. Gene dose reduction by heterozygous deletion of a master transcription factor and inactivating mutations in its coactivators impairs the activation component of differentiation genes epigenetically^6^. Aberrant amplifications in transcriptional corepressor enzymes facilitate a closed chromatin status and epigenetically silence hundreds of differentiation genes^6, 7^ (Table 1). This mode of alteration is clinically relevant and can be developed to suppress proliferation even in *TP53* mutant malignancies^102, 105^

For exponential proliferation to occur decoupled from forward-differentiation, a high degree of corepressor activity is necessary for epigenetic silencing of lineage-differentiation genes. Consequently, aberrant corepressor activity is frequently observed in malignant cells, where hundreds of terminal differentiation genes have accumulation of active corepressors^6,89^. Unlike coactivators, which are frequently inactivated by genetic mutations/deletions^6^, corepressors are frequently either wild-type or amplified in malignant cells (Table 1; Fig. 4b). DNA methyl transferase 1 enzyme (DNMT1) is a corepressor for master TF and also the maintenance methyltransferase that recapitulates CpG methylation onto the newly synthesized DNA strand as cells go through cycles of division^91–93^. In TCGA PANCAN data, high levels of DNMT1 are associated with poor survival (*p* < 0.00001, *n* = 5145), compared to cases with low DNMT1 levels (*n* = 5199) (Fig. 5a). This suggests an important role of this enzyme in numerous human cancers. Therefore, multiple studies have evolved in the last decade attempting to develop therapeutic interventions targeting DNMT1 in cancer therapy^94–102^. Similarly, Ubiquitin-like, containing PHD and RING finger domains, 1, (UHRF1), closely cooperates with DNMT1 in regulating DNA methylation^103,104^. We analyzed the expression levels of UHRF1 in the PANCAN dataset and found that high UHRF1 expression levels (*p* < 0.0001, *n* = 5150) strongly predicted poor survival rates compared to low levels (*n* = 5189) (Fig. 5b), illustrating the importance of these methylation genes in human cancers.

DNMT1-depletion without cytotoxicity has therapeutic benefits even in myelodysplastic syndrome (MDS) and acute myeloid leukemia (AML) containing p53-system defects^102,105^, and multiple clinical trials are ongoing to evaluate DNMT1-depletion more broadly in cancer therapy (although decitabine and 5-azacytidine used to deplete DNMT1 have pharmacologic limitations which can undermine their ability to deplete-DNMT1 from solid tumors) (Table 2). In acute promyelocytic leukemia (APL), complete remissions are achieved by combination of arsenic with retinoic acid to inhibit corepressors recruited on leukemia fusion protein PML-RARA^106,107^. Since co-repressors are not mutated and have aberrant activity in cancer, they are sufficient and logical molecular targets that may engage terminal differentiation genes for p53 cell cycle exits^7,89,99,100,102,105,108–111^ (Table 2; Fig. 5c, d).

**Table 2:** Key pre-clinical and clinical evaluation of corepressor therapy in cancer

Drugs	Disease	Dosage	Status	Survival: Ctrl vs. (Tx)	Adverse effects	Ref.
*Corepressor therapy in pre-clinical trials*						
5-aza-Dc	Ovarian cancer (in-vitro Ovarian cancer cell lines)	0.1–10 µM	Complete	No survival, decreased cell proliferation	None reported	^119^
THU* + Decitabine (Increased decitabine activity in the liver)	AML engrafted in the liver (mouse)	0.1–0.2 mg/kg	Complete	Median 38 days (61days)	None reported	^109^
Guadecitabine	HCC (HCC xenografts)	2 mg/Kg	Complete	Reduced tumor size	None reported	^120^
Decitabine	PDAC (mouse)	1 μg/g of body weight	Complete	Median 87 days (127.5 days)	None reported	^101^
*Co-repressor therapy in human clinical trials*						
Drugs	Disease	Dosage	Status	% ORR (%CR)	Adverse effects	Ref
Decitabine	Ovarian cancer	10–20 mg/m^2^	complete	60(10)	4 neutropenia at 20 mg/m^2^, none at 10 mg/m^2^	^121^
Decitabine	Advanced HCC	6 mg/m^2^/d	Complete	47(6)	Favorable adverse events	^122^
Decitabine + THU	Pancreatic cancer	Dec(5 mg/capsule) THU (250 mg/ capsule)	Recruiting	Not available at this time	Not available at this time	Clinicaltrial.gov
Decitabine + THU	NSCLC Lung cancer	THU 10mg/kg decitabine 0.2 mg/kg	Recruiting	Not available at this time	Not available at this time	Clinicaltrial.gov
Decitabine	MDS	0.1–0.2 mg/kg/day	Complete	44(55)	Neutropenic fever	^102^

Multiple preclinical studies evaluating mechanisms of non-cytotoxic therapy that engages differentiation genes instead of apoptosis genes show the proof principle for corepressor inhibition therapy across multiple tumors by targeting the corepressor DNMT1. Emerging human clinical trials are now evaluating corepressor therapies as p53 independent treatments in cancer therapy

*THU* tetrahydrouridine, *5-aza-Dc* 5-aza-deoxycytadine, *ORR* overall response rate, *CR* complete response, *TX* treatment group, *ctrl* control group, *ref* references

Various other corepressors have also been investigated as potential molecular targets for epigenetic therapy of cancer. For instance, histone deacetylase (HDAC) enzymes are key corepressors recruited into TF hubs of many human malignancies and are known epigenetic suppressors of gene expression^5,6,88,89^. In many pre-clinical studies, HDAC enzymes have been investigated as potential inducers of cell differentiation^94,95^. One problem with targeting HDACs, however, is their pleiotropic cellular functions—even on-target activity may thus produce unintended side effects. Other common corepressors upregulated in many human malignancies are lysine demethylase enzymes such as KDM1A (Fig. 4b). Various studies have demonstrated differentiation induction by pharmacologic targeting of KDM1A and related clinical trials are currently ongoing^112–116^. Using a high throughput pan-cancer in vivo screen, Carugo et al. recently demonstrated a link between the corepressor WDR5 and sustained MYC mediated proliferation of PDAC^117^. Disrupting WDR5 through inhibition assays led to arrested tumor progression and increased survival in PDX mouse models of PDAC^117^. In this systematic review, we have documented additional corepressors recruited into the master TF hubs of many human malignancies that require additional genetic and pharmacologic validation as candidate molecular targets that enhance differentiation. These include HES1, BAZ1A/B, BAZ2A, EED, SUZ12 and UHRF1 (Figs. 4b, 5b; Table 1). Furthermore, upregulation of these corepressors was found linked to advanced clinical pathological stages suggesting direct effect on differentiation suppression. For example, HES1 was found as the most frequently upregulated corepressor in OVC (Fig. 4b), and stage III and IV OVC had higher HES1 gains compared to stages I and II (Fig. 4c). Thus, HES1 inhibition therapy may be vital for OVC differentiation therapy. This pattern was also seen for BAZ1B in PDAC and KDM1B in HCC (Fig. 4b, d, e). These observations suggest that, in these malignancies, targeting these key enzymes for differentiation induction could provide additional therapeutic strategies that circumvent p53-system defects.

## Discussion

Human malignancies upregulate the master regulator of cell proliferation MYC, and this genetic alteration is significantly linked to poor survival rates. Historically, MYC-driven malignant proliferation has been antagonized by induction of apoptosis. Malignant cells, however, often harbor inactivating alterations to the master regulator of apoptosis p53 or its key co-factors, resulting in multi-drug resistance but continued apoptosis-induction in normal dividing cells (poor therapeutic index)^7,43,56^. To improve on issues of resistance and toxicity, it is thus imperative to find p53-independent strategies for antagonizing MYC^6,7^. Restoring forward-differentiation is one such potential strategy, and can be guided by an understanding that forward-differentiation is suppressed by partial loss-of-function to master TF that drive lineage-fates and to the coactivators they use to activate lineage-differentiation genes. This results in unbalanced activity of corepressors that repress the lineage-genes instead^33,34,76,89^. The corepressors are not frequently inactivated in human malignancies but are upregulated by CN gains and amplification of their chromosomal segments. Therefore, targeting these corepressors can provide therapy that engages differentiation instead of apoptosis^7,88,89,99,101,102,105^. Various clinical trials targeting transcriptional corepressors (e.g., DNMT1) without cytotoxicity have produced meaningful clinical responses^102,105^.

To sustain a given tissue through daily wear and tear, tissue lineage-progenitors proliferate exponentially^2,39^. Since cell division involves mechanical processes such as DNA replication, mitosis and meiosis, that are prone to error^2,4,9^, metazoan cells contain an apoptosis program to ensure that only healthy cells continue through phases of the cell cycle^44,45^. Therefore, p53/apoptosis potently antagonizes MYC to halt proliferation.

The 2012 Nobel prize awarded to Yamanaka and colleagues demonstrated in spectacular fashion that cell lineage-fates are commanded by handfuls of master TFs^68,74^. Master TFs combine and collaborate to exchange corepressors (“off enzymes”) for coactivators (“on enzymes”) and activate lineage-programs^6,7,118^. It is clear from the broadly available genomic data on cancers (TCGA etc.) that lineage master TFs, and the coactivators they use to activate target genes, are very frequently haploinsufficient in cancers. Crucially, however, unlike p53 or p16, these master TF circuits are not completely inactivated, and small molecule drugs that target the transcriptional corepressors aberrantly enriched in these highly expressed master TF hubs can resume forward lineage-differentiation and terminate proliferation, even if p53 or p16 are absent^7,89,99,100,102,105,109^. Importantly, such treatments simultaneously spare normal tissue stem cells needed for health and life.

## Conclusion and perspective

The enduring problems of oncotherapy are resistance and toxicity. A major reason for this situation is that most oncotherapeutics are designed to induce apoptosis, yet, the master regulators of this program are very frequently bi-allelically inactivated in cancers^11,43^. An alternative pathway for terminating malignant self-replication is to re-engage forward-differentiation in these differentiation-arrested cells. Pre-clinical genetic, epigenetic, biochemical and cell data, and both pre-clinical and clinical in vivo data, suggests that this can be achieved by inhibiting the corepressors aberrantly enriched, at the expense of coactivators, in the lineage master TF hubs highly expressed in replicating cancer cells.

## Electronic supplementary material

Prisma flow diagram

Disease free and overall survival comparisons in cancers with TP53/CDKN2A high versus low Frequency alterations
