# Supplementary figures and images for: Differentiation therapy and the mechanisms that terminate cancer cell proliferation without harming normal cells
Source: Cell Death Dis. 2018 Sep 6;9(9):912. doi: 10.1038/s41419-018-0919-9 (PMC6127320; doi:10.1038/s41419-018-0919-9)

## Flow diagram

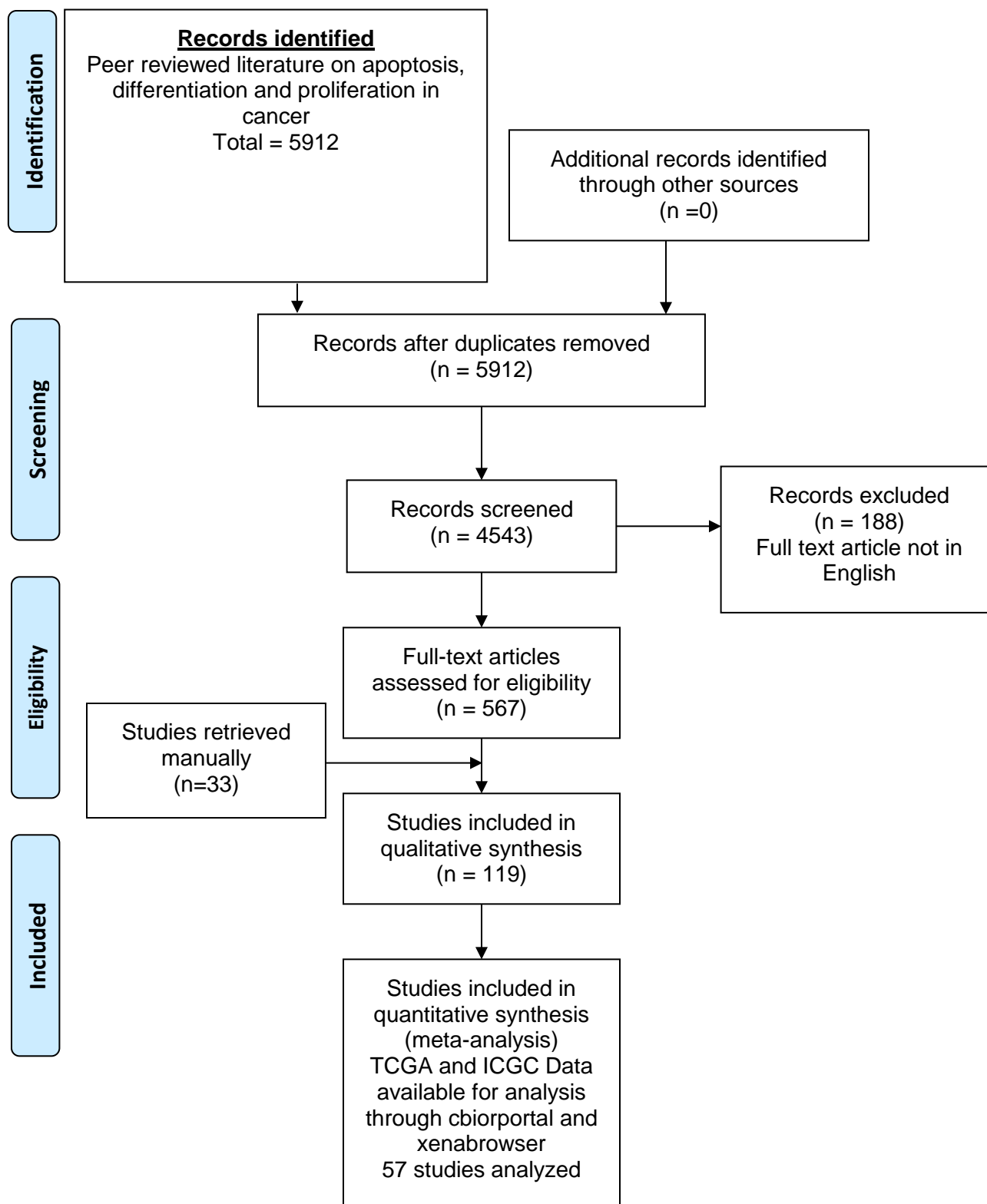

Supplement: Supplementary file 1 — Prisma flow diagram [file 41419_2018_919_MOESM1_ESM.pdf]
