# Supplementary material for: Differentiation therapy and the mechanisms that terminate cancer cell proliferation without harming normal cells
Source: Cell Death Dis. 2018 Sep 6;9(9):912. doi: 10.1038/s41419-018-0919-9 (PMC6127320; doi:10.1038/s41419-018-0919-9)
Supplement: Supplementary file 2 — Disease free and overall survival comparisons in cancers with TP53/CDKN2A high versus low Frequency alterations [file 41419_2018_919_MOESM2_ESM.docx]

**TP53/CDKN2A High**

| **Liver cancer (HCC) TCGA provisional dataset** | | | | |
| --- | --- | --- | --- | --- |
| **Overall survival** | Total (n) | Deceased (n) | Median mo. Survival | LogRank pvalue |
| Cases with Alteration(s) in TP53 & CDKN2A | 141 | 53 | 55.35 | 0.0294 |
| Cases without Alteration(s) in TP53 & CDKN2A | 224 | 74 | 69.51 |  |
| **Progression free survival** | Total (n) | Deceased (n) | Median mo. Disease free | LogRank pvalue |
| Cases with Alteration(s) in TP53 & CDKN2A | 122 | 74 | 12.61 | 0.0044 |
| Cases without Alteration(s) in TP53 & CDKN2A | 192 | 97 | 27.2 |  |
| **Lung carcinoma TCGA Provisional dataset** | | | | |
| **Overall survival** | Total (n) | Deceased (n) | Median mo. Survival | LogRank pvalue |
| Cases with Alteration(s) in TP53 & CDKN2A | 130 | 57 | 39.32 | 0.0263 |
| Cases without Alteration(s) in TP53 & CDKN2A | 91 | 34 | 53.29 |  |
| **Progression free survival** | Total (n) | Deceased (n) | Median mo. Disease free | LogRank pvalue |
| Cases with Alteration(s) in TP53 & CDKN2A | 106 | 51 | 35.81 | 0.557 |
| Cases without Alteration(s) in TP53 & CDKN2A | 80 | 38 | 41.23 |  |
| **Pancreatic ductal adenocarcinoma (PDAC) TCGA provisional dataset** | | | | |
| **Overall survival** | Total (n) | Deceased (n) | Median mo. Survival | LogRank pvalue |
| Cases with Alteration(s) in TP53 & CDKN2A | 122 | 72 | 19.48 | 0.0647 |
| Cases without Alteration(s) in TP53 & CDKN2A | 27 | 10 | 71.68 |  |
| **Progression free survival** | Total (n) | Deceased (n) | Median mo. Disease free | LogRank pvalue |
| Cases with Alteration(s) in TP53 & CDKN2A | 94 | 65 | 13.67 | 0.0007996 |
| Cases without Alteration(s) in TP53 & CDKN2A | 21 | 6 | 52.56 |  |
| **Ovarian cancer (OVC) TCGA provisional dataset** | | | | |
| **Overall survival** | Total (n) | Deceased (n) | Median mo. Survival | LogRank pvalue |
| Cases with Alteration(s) in TP53 & CDKN2A | 302 | 199 | 45.11 | 0.266 |
| Cases without Alteration(s) in TP53 & CDKN2A | 7 | 6 | 34.13 |  |
| **Progression free survival** | Total (n) | Deceased (n) | Median mo. Disease free | LogRank pvalue |
| Cases with Alteration(s) in TP53 & CDKN2A | 258 | 205 | 16.33 | 0.555 |
| Cases without Alteration(s) in TP53 & CDKN2A | 5 | 5 | 18.96 |  |
| **Glioblastoma (GBM) TCGA provisional dataset** | | | | |
| **Overall survival** | Total (n) | Deceased (n) | Median mo. Survival | LogRank pvalue |
| Cases with Alteration(s) in TP53 & CDKN2A | 222 | 150 | 12.9 | 0.0328 |
| Cases without Alteration(s) in TP53 & CDKN2A | 24 | 13 | 19.7 |  |
| **Progression free survival** | Total (n) | Deceased (n) | Median mo. Disease free | LogRank pvalue |
| Cases with Alteration(s) in TP53 & CDKN2A | 223 | 117 | 8.4 | 0.609 |
| Cases without Alteration(s) in TP53 & CDKN2A | 24 | 13 | 13.1 |  |
| **Esophageal Carcinoma TCGA provisional dataset** | | | | |
| **Overall survival** | Total (n) | Deceased (n) | Median mo. Survival | LogRank pvalue |
| Cases with Alteration(s) in TP53 & CDKN2A | 162 | 67 | 26.31 | 0.509 |
| Cases without Alteration(s) in TP53 & CDKN2A | 22 | 9 | 25.76 |  |
| **Progression free survival** | Total (n) | Deceased (n) | Median mo. Disease free | LogRank pvalue |
| Cases with Alteration(s) in TP53 & CDKN2A | 125 | 63 | 21.42 | 0.538 |
| Cases without Alteration(s) in TP53 & CDKN2A | 17 | 7 | 18 |  |
| **Bladder cancer TCGA provisional dataset** | | | | |
| **Overall survival** | Total (n) | Deceased (n) | Median mo. Survival | LogRank pvalue |
| Cases with Alteration(s) in TP53 & CDKN2A | 98 | 51 | 23.19 | 0.415 |
| Cases without Alteration(s) in TP53 & CDKN2A | 28 | 10 | 32 |  |
| **Progression free survival** | Total (n) | Deceased (n) | Median mo. Disease free | LogRank pvalue |
| Cases with Alteration(s) in TP53 & CDKN2A | 75 | 41 | 19.05 | 0.993 |
| Cases without Alteration(s) in TP53 & CDKN2A | 24 | 10 | 17.94 |  |
| **Breast cancer TCGA provisional dataset** | | | | |
| **Overall survival** | Total (n) | Deceased (n) | Median mo. Survival | LogRank pvalue |
| Cases with Alteration(s) in TP53 & CDKN2A | 328 | 58 | 212.09 | 0.0228 |
| Cases without Alteration(s) in TP53 & CDKN2A | 633 | 77 | 129.47 |  |
| **Progression free survival** | Total (n) | Deceased (n) | Median mo. Disease free | LogRank pvalue |
| Cases with Alteration(s) in TP53 & CDKN2A | 294 | 42 | NA | 0.0644 |
| Cases without Alteration(s) in TP53 & CDKN2A | 585 | 60 | 168.1 |  |
| **Lung adenocarcinoma TCGA provisional dataset** | | | | |
| **Overall survival** | Total (n) | Deceased (n) | Median mo. Survival | LogRank pvalue |
| Cases with Alteration(s) in TP53 & CDKN2A | 130 | 57 | 39.32 | 0.0263 |
| Cases without Alteration(s) in TP53 & CDKN2A | 91 | 34 | 53.29 |  |
| **Progression free survival** | Total (n) | Deceased (n) | Median mo. Disease free | LogRank pvalue |
| Cases with Alteration(s) in TP53 & CDKN2A | 106 | 51 | 35.81 | 0.557 |
| Cases without Alteration(s) in TP53 & CDKN2A | 80 | 38 | 41.23 |  |
| **NSCLC TCGA provisional dataset** | | | | |
| **Overall survival** | Total (n) | Deceased (n) | Median mo. Survival | LogRank pvalue |
| Cases with Alteration(s) in TP53 & CDKN2A | 130 | 57 | 39.32 | 0.0263 |
| Cases without Alteration(s) in TP53 & CDKN2A | 91 | 34 | 53.29 |  |
| **Progression free survival** | Total (n) | Deceased (n) | Median mo. Disease free | LogRank pvalue |
| Cases with Alteration(s) in TP53 & CDKN2A | 106 | 51 | 35.81 | 0.557 |
| Cases without Alteration(s) in TP53 & CDKN2A | 80 | 38 | 41.23 |  |

**TP53/CDKN2A Low**

| **Testicular cancer TCGA provisional dataset** | | | | |
| --- | --- | --- | --- | --- |
| **Overall survival** | Total (n) | Deceased (n) | Median mo. Survival | LogRank pvalue |
| Cases with Alteration(s) in TP53 & CDKN2A | 3 | 0 | NA | 0.804 |
| Cases without Alteration(s) in TP53 & CDKN2A | 130 | 4 | NA |  |
| **Progression free survival** | Total (n) | Deceased (n) | Median mo. Disease free | LogRank pvalue |
| Cases with Alteration(s) in TP53 & CDKN2A | 3 | 1 | 16.1 | 0.553 |
| Cases without Alteration(s) in TP53 & CDKN2A | 128 | 32 | 191.3 |  |
| **Cervical TCGA provisional dataset** | | | | |
| **Overall survival** | Total (n) | Deceased (n) | Median mo. Survival | LogRank pvalue |
| Cases with Alteration(s) in TP53 & CDKN2A | 11 | 2 | NA | 0.505 |
| Cases without Alteration(s) in TP53 & CDKN2A | 180 | 39 | 101.74 |  |
| **Progression free survival** | Total (n) | Deceased (n) | Median mo. Disease free | LogRank pvalue |
| Cases with Alteration(s) in TP53 & CDKN2A | 11 | 2 | NA | 0.891 |
| Cases without Alteration(s) in TP53 & CDKN2A | 162 | 29 | NA |  |
| **Prostate cancer TCGA provisional dataset** | | | | |
| **Overall survival** | Total (n) | Deceased (n) | Median mo. Survival | LogRank pvalue |
| Cases with Alteration(s) in TP53 & CDKN2A | 94 | 4 | NA | 0.44 |
| Cases without Alteration(s) in TP53 & CDKN2A | 404 | 6 | 115.05 |  |
| **Progression free survival** | Total (n) | Deceased (n) | Median mo. Disease free | LogRank pvalue |
| Cases with Alteration(s) in TP53 & CDKN2A | 92 | 26 | 666.89 | 0.0276 |
| Cases without Alteration(s) in TP53 & CDKN2A | 400 | 65 | NA |  |
| **Kidney Renal cell clear carcinoma TCGA provisional dataset** | | | | |
| **Overall survival** | Total (n) | Deceased (n) | Median mo. Survival | LogRank pvalue |
| Cases with Alteration(s) in TP53 & CDKN2A | 33 | 19 | 39.42 | 0.000 |
| Cases without Alteration(s) in TP53 & CDKN2A | 415 | 133 | 116.75 |  |
| **Progression free survival** | Total (n) | Deceased (n) | Median mo. Disease free | LogRank pvalue |
| Cases with Alteration(s) in TP53 & CDKN2A | 23 | 11 | 46.85 | 0.0271 |
| Cases without Alteration(s) in TP53 & CDKN2A | 343 | 103 | 123.72 |  |
| **Uterine corpus endometrial carcinoma TCGA provisional dataset** | | | | |
| **Overall survival** | Total (n) | Deceased (n) | Median mo. Survival | LogRank pvalue |
| Cases with Alteration(s) in TP53 & CDKN2A | 69 | 17 | 110.55 | 0.0033 |
| Cases without Alteration(s) in TP53 & CDKN2A | 173 | 15 | NA |  |
| **Progression free survival** | Total (n) | Deceased (n) | Median mo. Disease free | LogRank pvalue |
| Cases with Alteration(s) in TP53 & CDKN2A | 63 | 22 | NA | 0.00663 |
| Cases without Alteration(s) in TP53 & CDKN2A | 165 | 24 | NA |  |
| **Thymoma TCGA provisional dataset** | | | | |
| **Overall survival** | Total (n) | Deceased (n) | Median mo. Survival | LogRank pvalue |
| Cases with Alteration(s) in TP53 & CDKN2A | 23 | 4 | NA | 0.0029 |
| Cases without Alteration(s) in TP53 & CDKN2A | 99 | 5 | NA |  |
| **Progression free survival** | Total (n) | Deceased (n) | Median mo. Disease free | LogRank pvalue |
| Cases with Alteration(s) in TP53 & CDKN2A | 20 | 5 | NA | 0.128 |
| Cases without Alteration(s) in TP53 & CDKN2A | 98 | 16 | NA |  |
| **Uveal TCGA provisional dataset** | | | | |
| **Overall survival** | Total (n) | Deceased (n) | Median mo. Survival | LogRank pvalue |
| Cases with Alteration(s) in TP53 & CDKN2A | 9 | 3 | NA | 0.0011 |
| Cases without Alteration(s) in TP53 & CDKN2A | 59 | 8 | NA |  |
| **Progression free survival** | Total (n) | Deceased (n) | Median mo. Disease free | LogRank pvalue |
| Cases with Alteration(s) in TP53 & CDKN2A | 20 | 5 | NA | 0.286 |
| Cases without Alteration(s) in TP53 & CDKN2A | 98 | 16 | NA |  |
| **AML TCGA provisional dataset** | | | | |
| **Overall survival** | Total (n) | Deceased (n) | Median mo. Survival | LogRank pvalue |
| Cases with Alteration(s) in TP53 & CDKN2A | 23 | 21 | 8.97 | 0.0006 |
| Cases without Alteration(s) in TP53 & CDKN2A | 154 | 93 | 18.96 |  |
| **Progression free survival** | Total (n) | Deceased (n) | Median mo. Disease free | LogRank pvalue |
| Cases with Alteration(s) in TP53 & CDKN2A | 2 | 0 | NA | NaN |
| Cases without Alteration(s) in TP53 & CDKN2A | 61 | 0 | NA |  |
| **Thyroid TCGA provisional dataset** | | | | |
| **Overall survival** | Total (n) | Deceased (n) | Median mo. Survival | LogRank pvalue |
| Cases with Alteration(s) in TP53 & CDKN2A | 20 | 2 | NA | 0.1440 |
| Cases without Alteration(s) in TP53 & CDKN2A | 329 | 12 | NA |  |
| **Progression free survival** | Total (n) | Deceased (n) | Median mo. Disease free | LogRank pvalue |
| Cases with Alteration(s) in TP53 & CDKN2A | 19 | 4 | 68.76 | 0.105 |
| Cases without Alteration(s) in TP53 & CDKN2A | 367 | 31 | NA |  |
| **Medulla blastoma (MBL) ICGC dataset** | | | | |
| **Overall survival** | Total (n) | Deceased (n) | Median mo. Survival | LogRank pvalue |
| Cases with Alteration(s) in TP53 & CDKN2A | 6 | 2 | NA | 0.6890 |
| Cases without Alteration(s) in TP53 & CDKN2A | 97 | 23 | NA |  |
| **Progression free survival** | Total (n) | Deceased (n) | Median mo. Disease free | LogRank pvalue |
| Cases with Alteration(s) in TP53 & CDKN2A | NA | NA | NA | NaN |
| Cases without Alteration(s) in TP53 & CDKN2A | NA | NA | NA |  |
